# Genetic Analysis Workshop 18 single-nucleotide variant prioritization based on protein impact, sequence conservation, and gene annotation

**DOI:** 10.1186/1753-6561-8-S1-S11

**Published:** 2014-06-17

**Authors:** Thomas Nalpathamkalam, Andriy Derkach, Andrew D Paterson, Daniele Merico

**Affiliations:** 1 The Centre for Applied Genomics, The Hospital for Sick Children, 101 College Street, M5G 1L7 Toronto, ON, Canada; 2 Program in Genetics and Genome Biology, The Hospital for Sick Children, 101 College Street, M5G 1L7 Toronto, ON, Canada; 3 Department of Statistics, University of Toronto, 100 St. George St., M5S 3G3 Toronto, ON, Canada; 4 Division of Biostatistics, Dalla Lana School of Public Health, 155 College Street, University of Toronto, M5T 3M7 Toronto, ON, Canada

## Abstract

Grouping variants based on gene mapping can augment the power of rare variant association tests. Weighting or sorting variants based on their expected functional impact can provide additional benefit. We defined groups of prioritized variants based on systematic annotation of Genetic Analysis Workshop 18 (GAW18) single-nucleotide variants; we focused on variants detected by whole genome sequencing, specifically on the high-quality subset presented in the genotype files. First, we divided variants between coding and noncoding. Coding variants are fewer than 1% of the total and are more likely to have a biological effect than noncoding variants. Coding variants were further stratified into protein changing and protein damaging groups based on the effect on protein amino acid sequence. In particular, missense variants predicted to be damaging, splice-site alterations, and stop gains were assigned to the protein damaging category. Impact of noncoding variants is more difficult to predict. We decided to rely uniquely on conservation: we combined (a) the mammalian phastCons Conserved Element and (b) the PhyloP score, which identify conserved intervals and the single-nucleotide position, respectively. This reduced the noncoding variants to a number comparable to coding variants. Finally, using gene structure definition from the widely used RefSeq database, we mapped variants to genes to support association tests that require collapsing rare variants to genes. Companion GAW18 papers used these variant priority groups and gene mapping; one of these paper specifically found evidence of stronger association signal for protein damaging variants.

## Background

Next generation sequencing (NGS) technology, especially the application of whole genome sequencing (WGS), poses a major challenge in terms of the number of variants to be analyzed [1,2]. Grouping variants based on gene mapping can augment the power of rare variant association tests [2]. In addition, association studies, as well as other applications such as the search for Mendelian disease genes [1] or the interpretation of individual genomes for personalized medicine [3], can benefit from variant prioritization methods.

We prioritized Genetic Analysis Workshop 18 (GAW18) single-nucleotide variants based on criteria previously described in the literature [1,2] and publicly available bioinformatics resources.

First, we divided variants into coding and noncoding. Coding variants are fewer than 1% of the total and are more likely than noncoding variants to have a biological effect because they can produce a change in the protein sequence. Noncoding variants are more abundant and are more difficult to assess for functional relevance; however, many genome-wide association studies' signals have been found in noncoding regions, likely in correspondence of regulatory sequences [4]. For this reason, we decided to keep the two types of variants separate, and we decided to comparatively evaluate the detection of association signals for coding and noncoding variants. Both coding and noncoding variants were stratified in two progressively more stringent groups. For coding variants, the groups were protein changing and protein damaging; for noncoding variants, the groups were medium conservation and high conservation. Whereas coding variants were stratified based on the type of change introduced in the protein sequence, noncoding variants were stratified using evolutionary conservation of genomic DNA sequence as a proxy of functional relevance. Finally, variants were mapped to genes based on the overlap with their transcript. Intergenic variants were mapped to the closest gene, and ad-hoc rules were applied to minimize the number of variants mapping to multiple genes.

The Methods section describes in detail the tools and rules used to generate primary annotation, sort variants into priority groups, and map them to genes. The Results section describes in detail the number of variants found for each group and the rationale for the specific settings used for priority group definitions; we also briefly summarize results from a companion paper [5] showing the presence of association signals for prioritized variants. The Discussion section takes a critical look at the prioritization strategy adopted in this work in the context of recent literature on variant prioritization, making a few recommendations for future improvements.

## Methods

### Variant coordinates and alleles

GAW18 single-nucleotide variants were extracted from genotype files (.geno.csv), corresponding to 464 sequenced individuals; variants were provided for odd chromosomes. We identified reference and alternate alleles by considering the nucleotide at corresponding positions in the human genome reference sequence. Multi-allelic variants were considered bi-allelic, by considering only the two most frequent alleles; although not ideal, this substantially simplifies the annotation pipeline. We used the human genome reference sequence hg19 GRCh37.

### Extraction of variant annotation from databases

Annovar [6] was used to (a) map variants to gene exons and coding sequence on the basis of the RefSeq database (based on hg19) and (b) classify variants as synonymous (i.e., not expected to alter the protein sequence), missense (i.e., amino acid changing), stop-loss, stop-gain, or expected splicing alterations (defined as presence of alternate alleles in the +/- 2-bp interval around intron/exon boundaries). In the presence of overlapping genomic regions (e.g., 3′ untranslated region (3′ UTR) of gene A overlapping with coding exon of gene B), Annovar follows predefined annotation precedence (e.g., coding exon has precedence over 3′ UTR); the precedence rules used by Annovar are logically consistent with our variant prioritization strategy, which assigns higher priority to coding variants disrupting the protein sequence. Annovar's gene mapping was parsed to minimize the number of variants mapping to multiple genes: (a) we removed 68 read-through transcripts (i.e., transcripts that span two genes), which may represent artifacts or have dubious functional relevance, and (b) for intergenic regions, we reported only the closest genes and we removed all genes at a distance greater than 200 kbp.

To further classify missense variants, we used two widely used predictors, SIFT [7] and Polyphen [8], and combined their scores using CONDEL [9], a tool for optimized consensus definition; in particular, SIFT version 2.1.0 and Polyphen version 4.0.3 predictions were generated for all possible variant positions. To define noncoding variant conservation groups, we used placental mammals phastConsElements [10] and PhyloP [11] scores obtained from UCSC (based on hg19 coordinates).

### Definition of variant priority groups

We categorized variants into two mutually exclusive groups, coding and noncoding. For coding variants, we defined three groups based on expected impact on protein sequence: coding, protein changing, and protein damaging groups. Correspondingly, for noncoding variants, we defined three groups based on conservation: noncoding, noncoding medium conservation, and noncoding high conservation. The groups were defined as follows.

Variants categorized by Annovar as "splicing" or "exonic" were assigned to the "coding" group; all other variants (categorized by Annovar as "ncRNA", "UTR3", "UTR5", "intronic", "upstream", "downstream", "intergenic") were assigned to the noncoding category. In addition, coding variants categorized by Annovar as "splicing" or "exonic, nonsynonymous", "exonic, stop-gain" and "exonic, stop-loss" were also assigned to the "protein changing" subgroup of "coding". Finally, "splicing", "exonic, missense" variants predicted "deleterious" by CONDEL (a subset of "exonic, nonsynonymous" variants) and "exonic, stop-gain" variants were assigned to the "protein damaging" subgroup of "protein changing".

Noncoding variants with mammalian phastCons Conserved Element score greater than 0 and mammalian PhyloP greater than 1 were assigned to the noncoding subgroup "medium conservation". The noncoding variants satisfying more stringent cutoffs on conservation (phastCons >400; PhyloP >1.5) were assigned to the "high conservation" subgroup.

## Results

### Variant prioritization strategy

**Figure 1:**
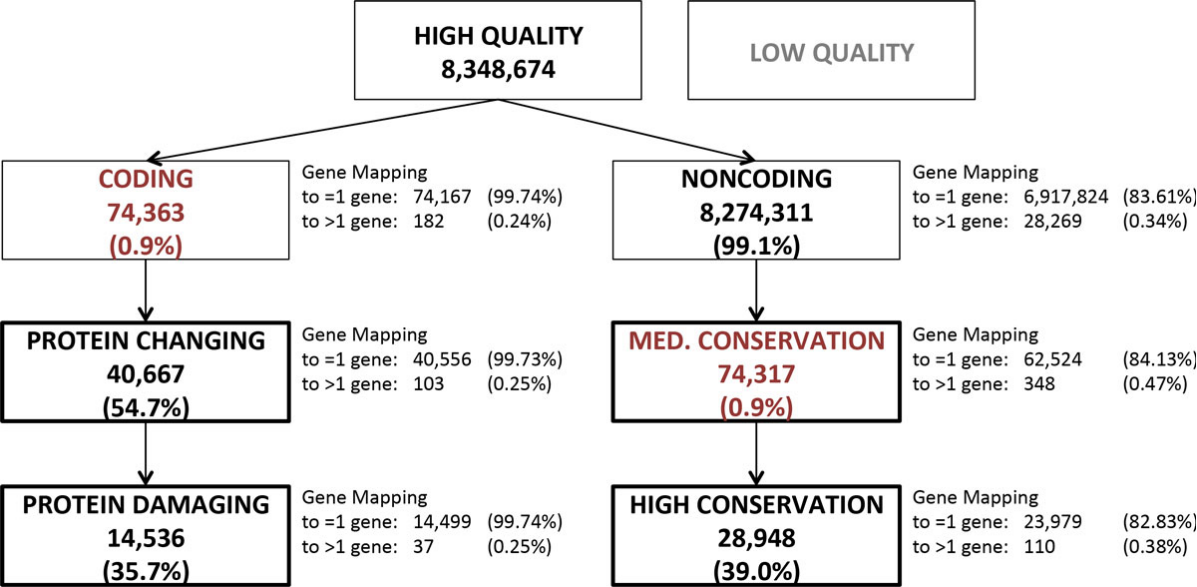
**Variant prioritization process and summary statistics**. Arrows represent the identification progressively of smaller groups of prioritized variants. Variant summary statistics are reported throughout the prioritization process; percentages in round brackets indicate the number of variants retained at each prioritization step. Variant groups expected to produce better association results have a thicker border line. Groups with a significant reduction in variant number are labeled in red.

We started from 8,348,674 high-quality single-nucleotide variants defined in the GAW18 genotype files. We decided to prioritize variants as groups rather than using a continuous ranking for sake of simplicity because several of the primary information items are binary (e.g., exonic: yes/no, stop-gain: yes/no). Variants were first sorted into two mutually exclusive groups (Figure 1), coding (74,363; 0.9%) and noncoding (8,274,311; 99.1%).

### Gene mappings

Gene mappings were parsed to remove mapping to read-through genes, which represent transcripts spanning two genes. Whereas some read-throughs are present in physiological conditions, but their functional role is dubious, other read-throughs are potential annotation artifacts. Read-throughs introduce extra redundancy when collapsing variants to genes, which we deemed unnecessary. After removing read-throughs, most of the coding variants (99.74%) mapped only to one gene. Gene mappings of intergenic variants were further processed by selecting only the closest gene and removing genes at a distance greater than 200 kbp; these rules could be regarded as simplistic, yet they help minimizing overlaps when collapsing rare variants by gene. This resulted in 83.6% of all noncoding variants mapping to one gene and only 0.34% mapping to more than one gene.

### Coding variant prioritization

Coding variants are a small minority, and they are more likely than noncoding variants to have a biological effect because they can produce a change in the protein sequence. In addition, there is a (at least partial) consensus on how to prioritize coding variants based on their protein sequence impact. Coding variants were defined as mapping to protein-coding exons as well as their splice sites; UTRs are not part of protein-coding exons and therefore were excluded. All other variants were assigned to the noncoding group regardless of their type (thus including UTRs and introns of protein-coding genes, ncRNA genes, promoters, and intergenic regions). Coding variants were labeled as protein changing (40,667; 54.7% of coding variants) if they altered splicing or if they changed at least one amino acid of the gene's protein sequence(s) (corresponding to the missense, stop-gain, and stop-loss categories). Protein changing variants were further labeled as protein damaging (14,536; 19.5%) if they altered splicing, if they introduced a stop codon, or if they produced an amino acid change deemed damaging by CONDEL [9], a missense variant impact consensus predictor that was used to integrate SIFT [7] and Polyphen [8] predictions. CONDEL weights predictions from these tools using a data set of 20,000 missense variants, both deleterious and neutral, and thus we deemed it superior to a simple consensus rule.

### Noncoding variant prioritization

**Figure 2:**
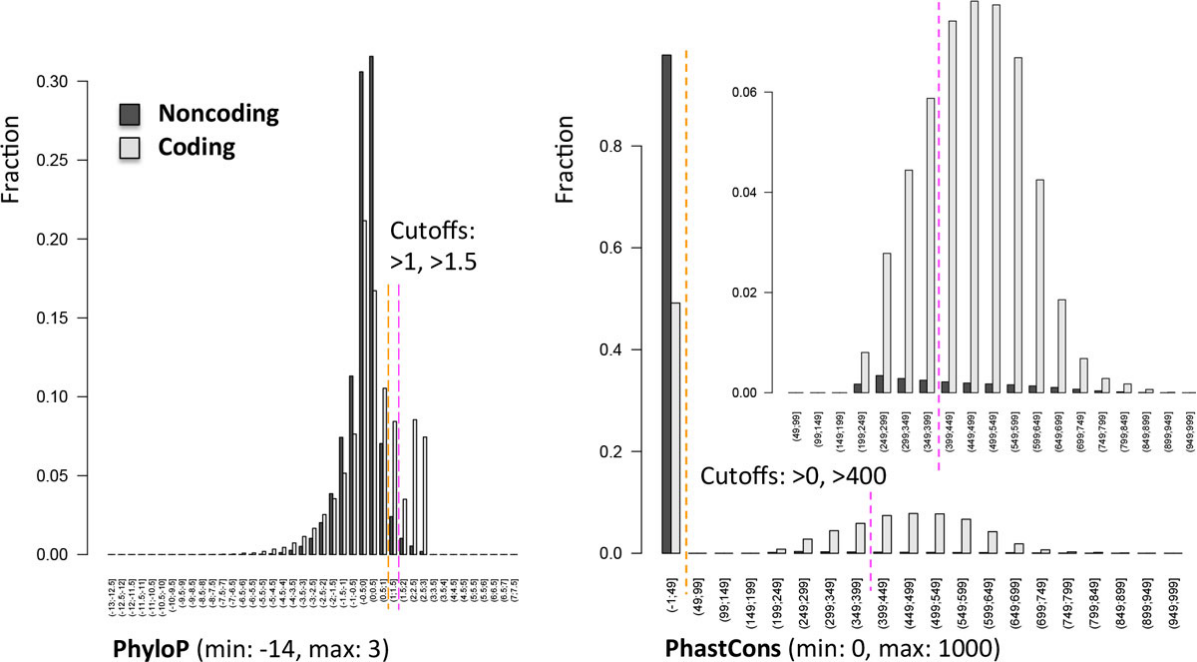
**Distribution of PhyloP and PhastCons scores**. Histograms of PhyloP (A) and PhastCons (B) scores across Genetic Analysis Workshop 18 for high quality coding (light gray) and noncoding (dark gray) variants. Orange and pink dashed lines indicate cutoffs used for medium and high conservation groups. For PhastCons, we also display a zoom over the distributions of score greater than 0.

To stratify noncoding variants, we used evolutionary conservation. In fact, for variants mapping to annotated noncoding RNAs or regulatory sequences (e.g., promoters, methylation sites), there is no established consensus on how to predict the functional disruption. In addition, many noncoding variants map to functionally relevant regions that have not yet been annotated [1]. At least some of the functionally relevant noncoding loci are likely to be constrained evolutionarily, and this method does depend on known annotations of functional elements. We defined two conservation priority groups based on two widely used conservation scores, mammalian phastCons Conserved Element [10] and PhyloP [11]. PhastCons score is based on a hidden Markov model that estimates the probability that each nucleotide belongs to a conserved element based on multiple alignment of genome sequences (placental mammal sequences in our case); PhastCons is sensitive to runs of conserved sites and is therefore effective at picking out conserved elements. By contrast, PhyloP separately measures conservation at individual positions, ignoring the conservation of neighboring positions. Variants with phastCons greater than 0 and PhyloP greater than 1 were assigned to the medium conservation group (74,317; 0.9% of all noncoding variants); variants with phastCons greater than 400 and PhyloP greater than 1.5 were assigned to the high conservation group (28,948; 39.0% of medium conservation noncoding variants). Well-characterized functional ncRNA elements (e.g., microRNAs) typically have phastCons greater than 400. We also verified that 54.5% of the coding variants reside within a phastCons greater than 0 genomic region, but only 2.4% of noncoding variants satisfied the same requirement (Figure 2), providing further support for the selected phastCons thresholds. The PhyloP cutoff was fine tuned to obtain a number of prioritized noncoding variant comparable to coding variants.

### Assessment of association for different priority groups

The GAW18 paper [5] assessed different gene-collapsed rare variant association test for 103 unrelated individuals with NGS data and real hypertension phenotype data, only for chromosome 3. None of the genes was significant after Bonferroni multiple test correction; however, quantile-quantile plots, investigating the discrepancy between real *p*-values and uniformly distributed *p*-values expected under the null hypothesis of no association, displayed an excess of nominally significant *p*-values for the protein damaging group compared with all coding variants. No signal was found for noncoding variants.

## Discussion

We defined a simple yet robust variant prioritization scheme using publicly available bioinformatics tools and resources. We first divided variants into coding and noncoding. Coding variants have a more straightforward mapping to genes and are capable of directly altering protein function. Nonetheless, at least some of the noncoding variants are expected to have a biologically relevant effect on functional noncoding RNA or protein-coding gene regulation. For both coding and noncoding variants, we defined a more inclusive and more stringent set of prioritized variants (coding: protein changing, protein damaging; noncoding: medium conservation and high conservation). We also provided gene mapping to support the grouping of variants by gene for association testing. These variant annotations were used in other GAW18 proceeding papers [5,12]; one paper [5] specifically found the presence of stronger association signals for protein damaging variants.

Several recent studies have investigated variants expected to alter protein function. In the following section, we summarize their findings and discuss their relevance for variant prioritization in association studies.

A study taking advantage of the large NHLBI-ESP exome variant database [13] has shown abundant rare and deleterious genetic variants, many of which arose recently in human evolutionary history because of accelerated population growth. Splice site alterations, stop-gain, and missense predicted-damaging variants were all shown to be on average more recent than synonymous variants, and a similar pattern was found for noncoding variants in conserved compared with nonconserved positions. This validates our overall strategy.

A survey of common loss-of-function (LOF) variants (including stop-gain and splice variants categorized as protein damaging in this study) [14] revealed several sources of error to be reckoned: sequencing or mapping errors (25.0%), annotation or reference sequence errors (26.8%), and variants unlikely to be genuine LOF events because of combined effects of other variants (11.1%). Excluding sequencing errors, which have been minimized in the GAW18 data set by additional quality filtering, these findings suggest that a more sophisticated annotation pipeline may be required to avoid false positive LOF variant annotation. This includes time-consuming manual inspection of all LOF variants, which was not practicable for this study, as well as more nuanced filters, such as considering the percentage of disrupted coding sequence and the presence of variant combinations that could cancel out the LOF in combination (only partially practicable in this study because in/del variants were not available).

We are not aware of specific works setting community-accepted standards for noncoding variants. Future work in this field will be extremely relevant to variant prioritization.

## Conclusions

We defined a simple yet robust variant prioritization scheme using publicly available bioinformatics tools and resources. We also provided gene mapping to support the grouping of variants by gene for association testing. Association signal was specifically found for coding variants predicted to be deleterious for protein function.

## Competing interests

The authors declare that they have no competing interests.

## Authors' contributions

DM and ADP designed the. TN and DM performed the data analysis. DM and ADP drafted the manuscript. TN and AD helped revise the manuscript. All authors read and approved the final manuscript.
